# Editorialets

**Published:** 1906-03

**Authors:** 


### Editorialets.

ARMY MEDICAL CORPS EXAMINATIONS.—Preliminary examinations for appointment of assistant surgeons in the army will be held on May 1 and July 31, 1906, at points to be hereafter designated.

Permission to appear for examination can be obtained upon application to the Surgeon General, U. S. Army, Washington, D. C., from whom full information concerning the examination can be procured. The essential requirements to securing an invitation are that the applicant shall be a citizen of the United States, shall be between 22 and 30 years of age, a graduate of a medical school legally authorized to confer the degree of doctor of medicine, shall be of good moral character and habits, and shall have had at least one year's hospital training or its equivalent in practice. The examinations will be held concurrently throughout the country at points where boards can be convened. Due consideration will be given to the localities from which applications are received, in order to lessen the traveling expenses of applicants as much as possible.

In order to perfect all necessary arrangements for the examinations of May 1st, applications must be complete and in possession of the Surgeon General on or before April 1st. Early attention is therefore enjoined upon all intended applicants.

There are at present twenty-five vacancies in the medical corps of the army.

---

MY DEAR SUBSCRIBERS: If you received a little reminder lately—please remit. I'm doing my d——t to give you a good "Red Back," and the Von Boeckmann-Jones Printing Company have a depraved appetite for checks.

---

DR. JULIAN C. FIELD, of Denison, Texas, died at his home in that city February 26th, ult., aged 65. Dr. Field was a Confederate surgeon; a graduate of the Medical Department of the (now) Tulane University, class of 1861, and a long time member of the Texas State Medical Association.

---

THE STATE MEDICAL ASSOCIATION'S committee on Legislation and Public Policy consists of President J. E. Gilcreest and Secretary I. C. Chase, ex-officers, and Ex-President J. T. Wilson, Sherman, and Drs. T. T. Jackson, San Antonio, and J. T. Turner, Terrell. The committee met recently and mapped out the campaign for medical legislation next session, and it will be submitted to the House of Delegates at the approaching annual meeting of the State Association at Fort Worth April 24-5-6. It is understood that a State Board of Health will not be asked for; but that efforts will be confined to a new medical practice act,—a mixed Board of Medical Examiners, and an anatomical bill.

---

THE PHYSICIANS of Tyler, Texas, have established a post-graduate medical school in that city. The Austin physicians are moving in a similar undertaking, with prospects of success. Other towns will follow the example. This is on the suggestion of Dr. McCormack.

---

THEY SAY (in remitting): "Just come on with the good old 'Red Back.' It is food and drink."—J. F. SCRUGGS, Creek, Texas.

"Let the 'Red Back' come and keep a-coming. Your address on 'Sentiment and Science' alone is worth the cost of five years' subscription."—T. C. BRASWELL, Cost, Texas.

"The 'Red Back' keeps me posted with reference to the Texas doctors; keep it coming. Enclosed is check for $4. Oklahoma is a great country, far ahead of Texas in medical societies and 'fine doctors'; still I like to hear from the boys 'from the forks of the creek.' Keep the 'Red Back' coming."—T. J. DODSON, Magnum, Okla.

---

THE THIRTY-EIGHTH ANNUAL MEETING of the State Medical Association will be held at Fort Worth—a three days' session—beginning April 24th, prox. A magnificent program has been arranged, social and scientific, and a rousing attendance is assured. All you fellows must lay down the shovel and the hoe and take up the fiddle and the bow, and let there be no more hard work for ye tired doctor that week. Let us meet and have a lovefeast. We will all be better, wiser and happier for it. Boom! went the cannon.

---

STATE HEALTH OFFICER OF TEXAS, DR. G. R. TABOR, with Mrs. Tabor, is touring Europe. He had a private audience with the Pope on January 11th. The Pope presented him with a silver medallion of his Holiness, as appreciation of his yellow fever work in Texas and Mexico. They are expected home April 1.

---

DIED.—Austin, Texas, February 12 (ult.), Dr. E. V. Hamilton, of Austin, aged 42. Dr. Hamilton was a native of Franklin county, Tennessee, and a graduate of Rush Medical College. He died suddenly, cause unknown. Dr. Hamilton was a much respected practitioner and a member of the State and local medical societies.

---

"JIMMIE IS A *Good* BOY, BUT HE *Will* STEAL."—"The *Journal of the American Medical Association* is the greatest medical journal in the world. * * * It represents the American medical profession, and as such is the champion of national reforms and worthy of our support."—*Dr. I. C. Chase's Editorial in Texas State Journal of Medicine,* February, 1906, page 290.

"It is unfortunate that the State journals can not have an illustrious example set them by the great *Journal of the A. M. A.* in the matter of clean advertising."—*Dr. I. C. Chase's Editorial in Texas State Journal of Medicine,* February, 1906, same page as above.

*Query:* Does a journal that carries unclean advertising "represent the American medical profession"?

---

AN OPENING FOR A CAPABLE DOCTOR.—

BANGS, TEXAS, February 2, 1906.

*Editor Texas Medical Journal, Austin, Texas:*

Bangs is a thriving little town ten miles west of Brownwood, on the Santa Fe railroad. It is surrounded by a fine farming country and is thickly settled with a very fine class of people. There is a good opening here for an up-to-date doctor. We want a first-class man and a first-class doctor.

Fraternally,

M. L. LANFORD.

---

IN THE treatment of intussusception the irrigation method should not be persisted in for too long a period, forty-eight hours being the maximum limit. The fluid should not be injected under high pressure, the irrigator not being suspended more than one and one-half or two feet above the patient.—*International Journal of Surgery.*

